# Bis(4-bromo­benzo­yl)(2,7-dimethoxy­naphthalene-1,8-di­yl)dimethanone

**DOI:** 10.1107/S1600536810001819

**Published:** 2010-01-20

**Authors:** Shoji Watanabe, Kosuke Nakaema, Toyokazu Muto, Akiko Okamoto, Noriyuki Yonezawa

**Affiliations:** a Department of Organic and Polymer Materials Chemistry, Tokyo University of Agriculture & Technology, Koganei, Tokyo 184-8588, Japan

## Abstract

In the title compound, C_26_H_18_Br_2_O_4_, the two 4-bromo­benzoyl groups at the 1- and 8-positions of the naphthalene ring system are *anti* to each other. The dihedral angle between the two benzene rings is 50.92 (14)°. The dihedral angles between the two benzene rings and the naphthalene ring system are 70.18 (11) and 74.98 (12)°. A weak inter­molecular C—H⋯O hydrogen bond exists between the methyl group and the carbonyl O atom.

## Related literature

For general background to the regioselective formation of *peri-*aroylnaphthalene compounds, see: Okamoto & Yonezawa (2009 ▶). For related structures, see: Mitsui *et al.* (2009 ▶); Nakaema *et al.* (2007 ▶, 2008 ▶); Watanabe *et al.* (2010 ▶).
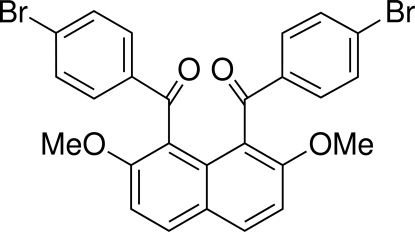


## Experimental

### 

#### Crystal data

C_26_H_18_Br_2_O_4_
*M* *_r_* = 554.22
Monoclinic, 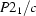

*a* = 7.8748 (5) Å
*b* = 25.7908 (16) Å
*c* = 11.5618 (7) Å
β = 100.982 (4)°
*V* = 2305.2 (2) Å^3^
*Z* = 4
Cu *K*α radiation
μ = 4.71 mm^−1^
*T* = 296 K
0.60 × 0.30 × 0.20 mm

#### Data collection

Rigaku R-AXIS RAPID diffractometer
Absorption correction: numerical (*NUMABS*; Higashi, 1999 ▶) *T*_min_ = 0.164, *T*_max_ = 0.452
42468 measured reflections
4220 independent reflections
3825 reflections with *I* > 2σ(*I*)
*R*_int_ = 0.064

#### Refinement

*R*[*F* ^2^ > 2σ(*F* ^2^)] = 0.038
*wR*(*F* ^2^) = 0.103
*S* = 1.05
4220 reflections
292 parameters
H-atom parameters constrained
Δρ_max_ = 0.73 e Å^−3^
Δρ_min_ = −0.66 e Å^−3^

### 

Data collection: *PROCESS-AUTO* (Rigaku, 1998 ▶); cell refinement: *PROCESS-AUTO*; data reduction: *CrystalStructure* (Rigaku/MSC, 2004 ▶); program(s) used to solve structure: *SIR2004* (Burla *et al.*, 2005 ▶); program(s) used to refine structure: *SHELXL97* (Sheldrick, 2008 ▶); molecular graphics: *ORTEPIII* (Burnett & Johnson, 1996 ▶); software used to prepare material for publication: *SHELXL97*.

## Supplementary Material

Crystal structure: contains datablocks I, global. DOI: 10.1107/S1600536810001819/is2516sup1.cif

Structure factors: contains datablocks I. DOI: 10.1107/S1600536810001819/is2516Isup2.hkl

Additional supplementary materials:  crystallographic information; 3D view; checkCIF report

## Figures and Tables

**Table 1:** Hydrogen-bond geometry (Å, °)

*D*—H⋯*A*	*D*—H	H⋯*A*	*D*⋯*A*	*D*—H⋯*A*
C25—H25*B*⋯O1^i^	0.96	2.42	3.313 (4)	155

Symmetry code: (i) 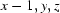.

**Fig. 1.**
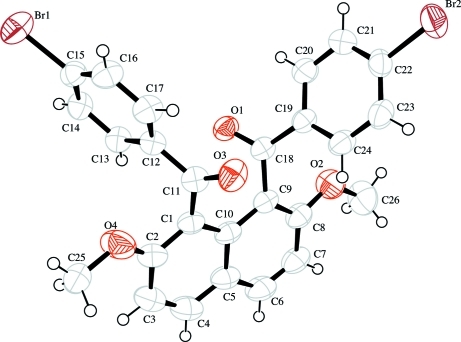
The molecular structure of the title compound with displacement ellipsoids at 50% probability for non-H atoms.

## supplementary crystallographic information

### Comment

In the course of our study on selective electrophilic aromatic aroylation of 2,7-dimethoxynaphthalene, *peri-*aroylnaphthalene compounds have proved to be formed regioselectively with the aid of suitable acidic mediator (Okamoto & Yonezawa, 2009). The aroyl groups at 1,8-positions of the naphthalene rings in these compounds are oriented in opposite fashion and are found to be non-coplanar resulting in partial disruption in π-conjugation systems. Recently, we have reported the X-ray crystal structures of 1,8-bis(4-chlorobenzoyl)-2,7-dimethoxynaphthalene (Nakaema *et al.*, 2007), 1,8-dibenzoyl-2,7-dimethoxynaphthalene (Nakaema *et al.*, 2008) and (2,7-dimethoxynaphthalene-1,8-diyl)-bis(4-fluorobenzoyl)dimethanone (Watanabe *et al.*, 2010). As a part of the course of our continuous study on the molecular structures of this kind of homologous molecules, the X-ray crystal structure of title compound, *peri*-aroylnaphthalene bearing bromo groups, is discussed in this report.

The molecular structure of the title molecule is displayed in Fig. 1. The two 4-bromobenzoyl groups are situated in *anti* orientation. Furthermore, these 4-bromobenzoyl groups are twisted away from the attached naphthalene ring. The interplanar angle between the best planes of two benzene rings is 50.92 (14)°. On the other hand, the two interplanar angles between the best planes of the *peri*-bromophenyl rings and the naphthalene ring are 70.18 (11) and 74.98 (12)°. The torsion angles between the carbonyl groups and the naphthalene ring are relatively large [C10—C1—C11—O3 = -53.3 (3)° and C10—C9—C18—O1 = -47.3 (3)°] and those between 4-bromophenyl groups and carbonyl groups are rather small [O3—C11—C12—C17 = -16.8 (4)° and O1—C18—C19—C20 = -20.0 (4)°]. The crystal packing is stabilized by weak C—H···O hydrogen bonds (Table 1).

### Experimental

The title compound was prepared by electrophilic aromatic diaroylation reaction of 2,7-dimethoxynaphthalene with 4-bromobenzoic acid. Colorless single crystals suitable for X-ray diffraction were obtained by recrystallization from ethanol.

Spectroscopic Data: ^1^H NMR (300 MHz, CDCl_3_): δ 3.69 (6H, s), 7.19 (2H, d, J = 9.0 Hz), 7.47 (4H, d, J = 8.4 Hz), 7.54 (4H, d, J = 8.4 Hz), 7.95 (2H, d, J = 9.0 Hz); ^13^C NMR (75.0 MHz, CDCl_3_): δ 56.3, 111.1, 120.6, 125.5, 127.9, 123.0, 130.6, 131.4, 132.5, 137.5, 156.5, 196.2; IR (KBr cm^-1^): 1660, 1269; m.p. = 250 °C.

### Refinement

All the H atoms were found in a difference map and were subsequently refined as riding, with C—H = 0.93 (aromatic) and 0.96 (methyl) Å, and with *U*_iso_(H) = 1.2*U*_eq_(C).

### Crystal data

**Table:**

C_26_H_18_Br_2_O_4_	*F*(000) = 1104
*M**_r_* = 554.22	*D*_x_ = 1.597 Mg m^−^^3^
Monoclinic, *P*2_1_/*c*	Melting point: 523 K
Hall symbol: -P 2ybc	Cu *K*α radiation, λ = 1.54187 Å
*a* = 7.8748 (5) Å	Cell parameters from 40803 reflections
*b* = 25.7908 (16) Å	θ = 3.4–68.2°
*c* = 11.5618 (7) Å	µ = 4.71 mm^−^^1^
β = 100.982 (4)°	*T* = 296 K
*V* = 2305.2 (2) Å^3^	Platelet, colorless
*Z* = 4	0.60 × 0.30 × 0.20 mm

### Data collection

**Table:**

Rigaku R-AXIS RAPID diffractometer	4220 independent reflections
Radiation source: rotating anode	3825 reflections with *I* > 2σ(*I*)
graphite	*R*_int_ = 0.064
Detector resolution: 10.00 pixels mm^-1^	θ_max_ = 68.2°, θ_min_ = 3.4°
ω scans	*h* = −9→9
Absorption correction: numerical (*NUMABS*; Higashi, 1999)	*k* = −31→31
*T*_min_ = 0.164, *T*_max_ = 0.452	*l* = −13→13
42468 measured reflections	

### Refinement

**Table:**

Refinement on *F*^2^	Secondary atom site location: difference Fourier map
Least-squares matrix: full	Hydrogen site location: difference Fourier map
*R*[*F*^2^ > 2σ(*F*^2^)] = 0.038	H-atom parameters constrained
*wR*(*F*^2^) = 0.103	*w* = 1/[σ^2^(*F*_o_^2^) + (0.0451*P*)^2^ + 1.8271*P*] where *P* = (*F*_o_^2^ + 2*F*_c_^2^)/3
*S* = 1.05	(Δ/σ)_max_ < 0.001
4220 reflections	Δρ_max_ = 0.73 e Å^−^^3^
292 parameters	Δρ_min_ = −0.66 e Å^−^^3^
0 restraints	Extinction correction: *SHELXL97* (Sheldrick, 2008), Fc^*^=kFc[1+0.001xFc^2^λ^3^/sin(2θ)]^-1/4^
Primary atom site location: structure-invariant direct methods	Extinction coefficient: 0.00195 (13)

### Special details

**Table:**

Geometry. All e.s.d.'s (except the e.s.d. in the dihedral angle between two l.s. planes) are estimated using the full covariance matrix. The cell e.s.d.'s are taken into account individually in the estimation of e.s.d.'s in distances, angles and torsion angles; correlations between e.s.d.'s in cell parameters are only used when they are defined by crystal symmetry. An approximate (isotropic) treatment of cell e.s.d.'s is used for estimating e.s.d.'s involving l.s. planes.
Refinement. Refinement of *F*^2^ against ALL reflections. The weighted *R*-factor *wR* and goodness of fit *S* are based on *F*^2^, conventional *R*-factors *R* are based on *F*, with *F* set to zero for negative *F*^2^. The threshold expression of *F*^2^ > σ(*F*^2^) is used only for calculating *R*-factors(gt) *etc*. and is not relevant to the choice of reflections for refinement. *R*-factors based on *F*^2^ are statistically about twice as large as those based on *F*, and *R*- factors based on ALL data will be even larger.

### Fractional atomic coordinates and isotropic or equivalent isotropic displacement parameters (Å^2^)

**Table:**

	*x*	*y*	*z*	*U*_iso_*/*U*_eq_	
Br1	0.72128 (5)	0.437493 (13)	0.07373 (3)	0.07441 (16)	
Br2	1.22096 (6)	0.071146 (15)	0.46717 (4)	0.08957 (19)	
O1	0.7621 (2)	0.18244 (7)	−0.02615 (16)	0.0540 (4)	
O2	0.7527 (3)	0.04184 (9)	−0.0577 (2)	0.0725 (6)	
O3	0.5203 (3)	0.18565 (8)	0.17194 (16)	0.0626 (5)	
O4	0.1847 (3)	0.25609 (10)	−0.0098 (2)	0.0817 (7)	
C1	0.3713 (3)	0.18756 (10)	−0.0245 (2)	0.0478 (6)	
C2	0.2137 (4)	0.21142 (12)	−0.0655 (3)	0.0591 (7)	
C3	0.0891 (4)	0.18868 (15)	−0.1549 (3)	0.0706 (8)	
H3	−0.0131	0.2061	−0.1852	0.085*	
C4	0.1205 (4)	0.14116 (15)	−0.1958 (3)	0.0711 (9)	
H4	0.0360	0.1256	−0.2523	0.085*	
C5	0.2764 (4)	0.11461 (12)	−0.1558 (2)	0.0585 (7)	
C6	0.3060 (5)	0.06490 (13)	−0.1987 (3)	0.0696 (9)	
H6	0.2184	0.0492	−0.2526	0.083*	
C7	0.4558 (5)	0.03943 (12)	−0.1646 (3)	0.0661 (8)	
H7	0.4694	0.0062	−0.1927	0.079*	
C8	0.5925 (4)	0.06332 (11)	−0.0859 (2)	0.0558 (7)	
C9	0.5715 (3)	0.11220 (10)	−0.0403 (2)	0.0460 (6)	
C10	0.4095 (3)	0.13854 (10)	−0.0715 (2)	0.0487 (6)	
C11	0.4846 (3)	0.21101 (10)	0.0821 (2)	0.0483 (6)	
C12	0.5453 (3)	0.26560 (10)	0.0768 (2)	0.0446 (5)	
C13	0.5458 (3)	0.29065 (10)	−0.0294 (2)	0.0474 (6)	
H13	0.5073	0.2731	−0.0999	0.057*	
C14	0.6028 (4)	0.34120 (10)	−0.0320 (2)	0.0508 (6)	
H14	0.6058	0.3576	−0.1032	0.061*	
C15	0.6551 (3)	0.36681 (10)	0.0736 (2)	0.0498 (6)	
C16	0.6569 (4)	0.34286 (11)	0.1802 (2)	0.0561 (7)	
H16	0.6937	0.3607	0.2504	0.067*	
C17	0.6036 (4)	0.29216 (11)	0.1813 (2)	0.0543 (6)	
H17	0.6066	0.2754	0.2530	0.065*	
C18	0.7312 (3)	0.14120 (10)	0.0171 (2)	0.0450 (5)	
C19	0.8499 (3)	0.12117 (9)	0.1232 (2)	0.0463 (6)	
C20	1.0174 (4)	0.14010 (12)	0.1497 (3)	0.0615 (7)	
H20	1.0549	0.1636	0.0990	0.074*	
C21	1.1292 (4)	0.12445 (13)	0.2506 (3)	0.0680 (8)	
H21	1.2420	0.1369	0.2677	0.082*	
C22	1.0712 (4)	0.09027 (10)	0.3249 (2)	0.0560 (7)	
C23	0.9078 (4)	0.07017 (11)	0.3003 (3)	0.0595 (7)	
H23	0.8718	0.0464	0.3511	0.071*	
C24	0.7965 (4)	0.08571 (11)	0.1986 (2)	0.0559 (7)	
H24	0.6851	0.0722	0.1809	0.067*	
C25	0.0244 (4)	0.28190 (14)	−0.0380 (4)	0.0872 (12)	
H25A	0.0302	0.3139	0.0050	0.105*	
H25B	−0.0647	0.2604	−0.0173	0.105*	
H25C	−0.0016	0.2890	−0.1210	0.105*	
C26	0.7792 (6)	−0.00847 (14)	−0.1026 (4)	0.0892 (12)	
H26A	0.8985	−0.0181	−0.0786	0.107*	
H26B	0.7494	−0.0080	−0.1870	0.107*	
H26C	0.7076	−0.0332	−0.0722	0.107*	

### Atomic displacement parameters (Å^2^)

**Table:**

	*U*^11^	*U*^22^	*U*^33^	*U*^12^	*U*^13^	*U*^23^
Br1	0.0966 (3)	0.0548 (2)	0.0726 (2)	−0.01775 (16)	0.01798 (19)	−0.00874 (14)
Br2	0.0988 (3)	0.0691 (3)	0.0839 (3)	0.00526 (19)	−0.0253 (2)	0.01543 (18)
O1	0.0562 (11)	0.0522 (11)	0.0536 (10)	−0.0098 (8)	0.0102 (8)	0.0071 (8)
O2	0.0782 (15)	0.0593 (12)	0.0778 (14)	0.0047 (11)	0.0087 (11)	−0.0213 (11)
O3	0.0877 (15)	0.0586 (11)	0.0417 (10)	−0.0066 (10)	0.0131 (9)	0.0028 (8)
O4	0.0507 (12)	0.0856 (16)	0.1094 (19)	0.0056 (11)	0.0163 (12)	−0.0257 (14)
C1	0.0456 (14)	0.0549 (15)	0.0445 (13)	−0.0100 (11)	0.0128 (10)	−0.0023 (11)
C2	0.0474 (15)	0.0674 (18)	0.0639 (17)	−0.0093 (13)	0.0136 (13)	−0.0031 (14)
C3	0.0493 (17)	0.089 (2)	0.070 (2)	−0.0042 (15)	0.0039 (14)	0.0004 (17)
C4	0.0564 (18)	0.089 (2)	0.0636 (18)	−0.0159 (16)	−0.0006 (14)	−0.0074 (17)
C5	0.0576 (16)	0.0679 (18)	0.0488 (14)	−0.0176 (14)	0.0072 (12)	−0.0061 (13)
C6	0.074 (2)	0.070 (2)	0.0597 (18)	−0.0244 (16)	0.0006 (15)	−0.0150 (14)
C7	0.083 (2)	0.0525 (16)	0.0622 (18)	−0.0176 (15)	0.0118 (16)	−0.0150 (14)
C8	0.0675 (18)	0.0517 (15)	0.0490 (15)	−0.0093 (13)	0.0129 (13)	−0.0054 (11)
C9	0.0539 (14)	0.0482 (13)	0.0366 (12)	−0.0111 (11)	0.0102 (10)	−0.0019 (10)
C10	0.0540 (15)	0.0542 (14)	0.0391 (12)	−0.0152 (12)	0.0121 (11)	−0.0001 (10)
C11	0.0513 (14)	0.0553 (15)	0.0407 (13)	−0.0019 (11)	0.0150 (11)	−0.0037 (11)
C12	0.0443 (13)	0.0524 (14)	0.0378 (12)	−0.0012 (10)	0.0096 (10)	−0.0031 (10)
C13	0.0558 (15)	0.0502 (14)	0.0361 (12)	0.0004 (11)	0.0082 (10)	−0.0064 (10)
C14	0.0608 (16)	0.0518 (14)	0.0406 (13)	0.0014 (12)	0.0116 (11)	0.0028 (11)
C15	0.0507 (14)	0.0465 (13)	0.0526 (14)	−0.0023 (11)	0.0104 (11)	−0.0047 (11)
C16	0.0661 (17)	0.0624 (17)	0.0396 (13)	−0.0110 (13)	0.0093 (12)	−0.0113 (12)
C17	0.0645 (17)	0.0627 (16)	0.0359 (12)	−0.0072 (13)	0.0103 (11)	−0.0021 (11)
C18	0.0501 (14)	0.0464 (13)	0.0406 (12)	−0.0057 (11)	0.0137 (10)	−0.0023 (10)
C19	0.0521 (14)	0.0437 (13)	0.0439 (13)	−0.0056 (10)	0.0113 (11)	−0.0026 (10)
C20	0.0567 (17)	0.0619 (17)	0.0647 (17)	−0.0107 (13)	0.0087 (13)	0.0141 (14)
C21	0.0543 (17)	0.0665 (19)	0.078 (2)	−0.0080 (14)	−0.0012 (15)	0.0080 (15)
C22	0.0661 (17)	0.0436 (14)	0.0532 (15)	0.0060 (12)	−0.0016 (13)	−0.0005 (11)
C23	0.078 (2)	0.0497 (15)	0.0505 (15)	−0.0066 (13)	0.0105 (14)	0.0055 (12)
C24	0.0618 (17)	0.0538 (15)	0.0515 (15)	−0.0148 (13)	0.0091 (12)	0.0022 (12)
C25	0.065 (2)	0.0543 (18)	0.142 (4)	0.0001 (15)	0.021 (2)	0.015 (2)
C26	0.107 (3)	0.068 (2)	0.089 (3)	0.018 (2)	0.009 (2)	−0.0228 (19)

### Geometric parameters (Å, °)

**Table:**

Br1—C15	1.896 (3)	C12—C17	1.389 (3)
Br2—C22	1.897 (3)	C13—C14	1.381 (4)
O1—C18	1.219 (3)	C13—H13	0.9300
O2—C8	1.359 (4)	C14—C15	1.381 (4)
O2—C26	1.427 (4)	C14—H14	0.9300
O3—C11	1.214 (3)	C15—C16	1.376 (4)
O4—C2	1.361 (4)	C16—C17	1.374 (4)
O4—C25	1.409 (4)	C16—H16	0.9300
C1—C2	1.386 (4)	C17—H17	0.9300
C1—C10	1.430 (4)	C18—C19	1.487 (4)
C1—C11	1.505 (4)	C19—C20	1.384 (4)
C2—C3	1.409 (4)	C19—C24	1.383 (4)
C3—C4	1.354 (5)	C20—C21	1.381 (4)
C3—H3	0.9300	C20—H20	0.9300
C4—C5	1.405 (5)	C21—C22	1.369 (4)
C4—H4	0.9300	C21—H21	0.9300
C5—C6	1.410 (4)	C22—C23	1.366 (4)
C5—C10	1.429 (4)	C23—C24	1.385 (4)
C6—C7	1.342 (5)	C23—H23	0.9300
C6—H6	0.9300	C24—H24	0.9300
C7—C8	1.412 (4)	C25—H25A	0.9600
C7—H7	0.9300	C25—H25B	0.9600
C8—C9	1.389 (4)	C25—H25C	0.9600
C9—C10	1.429 (4)	C26—H26A	0.9600
C9—C18	1.504 (3)	C26—H26B	0.9600
C11—C12	1.492 (4)	C26—H26C	0.9600
C12—C13	1.389 (3)		
			
C8—O2—C26	118.4 (3)	C13—C14—H14	120.8
C2—O4—C25	120.9 (3)	C16—C15—C14	121.9 (2)
C2—C1—C10	120.1 (2)	C16—C15—Br1	118.37 (19)
C2—C1—C11	117.0 (2)	C14—C15—Br1	119.8 (2)
C10—C1—C11	122.1 (2)	C17—C16—C15	119.0 (2)
O4—C2—C1	115.7 (3)	C17—C16—H16	120.5
O4—C2—C3	122.9 (3)	C15—C16—H16	120.5
C1—C2—C3	121.3 (3)	C16—C17—C12	120.8 (2)
C4—C3—C2	119.0 (3)	C16—C17—H17	119.6
C4—C3—H3	120.5	C12—C17—H17	119.6
C2—C3—H3	120.5	O1—C18—C19	119.9 (2)
C3—C4—C5	122.1 (3)	O1—C18—C9	117.9 (2)
C3—C4—H4	118.9	C19—C18—C9	122.2 (2)
C5—C4—H4	118.9	C20—C19—C24	119.0 (3)
C4—C5—C6	121.3 (3)	C20—C19—C18	118.9 (2)
C4—C5—C10	119.7 (3)	C24—C19—C18	122.1 (2)
C6—C5—C10	118.9 (3)	C21—C20—C19	120.7 (3)
C7—C6—C5	122.5 (3)	C21—C20—H20	119.6
C7—C6—H6	118.8	C19—C20—H20	119.6
C5—C6—H6	118.8	C22—C21—C20	118.9 (3)
C6—C7—C8	119.7 (3)	C22—C21—H21	120.6
C6—C7—H7	120.2	C20—C21—H21	120.6
C8—C7—H7	120.2	C23—C22—C21	121.8 (3)
O2—C8—C9	116.8 (2)	C23—C22—Br2	119.2 (2)
O2—C8—C7	122.4 (3)	C21—C22—Br2	119.0 (2)
C9—C8—C7	120.6 (3)	C22—C23—C24	119.1 (3)
C8—C9—C10	120.1 (2)	C22—C23—H23	120.5
C8—C9—C18	117.9 (2)	C24—C23—H23	120.5
C10—C9—C18	120.4 (2)	C19—C24—C23	120.5 (3)
C5—C10—C9	118.1 (2)	C19—C24—H24	119.8
C5—C10—C1	117.4 (3)	C23—C24—H24	119.8
C9—C10—C1	124.5 (2)	O4—C25—H25A	109.5
O3—C11—C12	121.3 (2)	O4—C25—H25B	109.5
O3—C11—C1	119.3 (2)	H25A—C25—H25B	109.5
C12—C11—C1	119.3 (2)	O4—C25—H25C	109.5
C13—C12—C17	118.9 (2)	H25A—C25—H25C	109.5
C13—C12—C11	122.1 (2)	H25B—C25—H25C	109.5
C17—C12—C11	119.0 (2)	O2—C26—H26A	109.5
C14—C13—C12	120.9 (2)	O2—C26—H26B	109.5
C14—C13—H13	119.5	H26A—C26—H26B	109.5
C12—C13—H13	119.5	O2—C26—H26C	109.5
C15—C14—C13	118.4 (2)	H26A—C26—H26C	109.5
C15—C14—H14	120.8	H26B—C26—H26C	109.5
			
C25—O4—C2—C1	−175.5 (3)	C10—C1—C11—O3	−53.2 (4)
C25—O4—C2—C3	1.0 (5)	C2—C1—C11—C12	−60.4 (3)
C10—C1—C2—O4	175.6 (2)	C10—C1—C11—C12	129.1 (3)
C11—C1—C2—O4	4.9 (4)	O3—C11—C12—C13	163.0 (3)
C10—C1—C2—C3	−1.0 (4)	C1—C11—C12—C13	−19.4 (4)
C11—C1—C2—C3	−171.6 (3)	O3—C11—C12—C17	−16.8 (4)
O4—C2—C3—C4	−172.1 (3)	C1—C11—C12—C17	160.8 (2)
C1—C2—C3—C4	4.1 (5)	C17—C12—C13—C14	−0.2 (4)
C2—C3—C4—C5	−2.7 (5)	C11—C12—C13—C14	−179.9 (2)
C3—C4—C5—C6	179.4 (3)	C12—C13—C14—C15	−1.7 (4)
C3—C4—C5—C10	−1.8 (5)	C13—C14—C15—C16	2.1 (4)
C4—C5—C6—C7	177.9 (3)	C13—C14—C15—Br1	−176.3 (2)
C10—C5—C6—C7	−1.0 (5)	C14—C15—C16—C17	−0.6 (4)
C5—C6—C7—C8	−2.2 (5)	Br1—C15—C16—C17	177.8 (2)
C26—O2—C8—C9	179.7 (3)	C15—C16—C17—C12	−1.3 (4)
C26—O2—C8—C7	−4.8 (5)	C13—C12—C17—C16	1.7 (4)
C6—C7—C8—O2	−173.1 (3)	C11—C12—C17—C16	−178.6 (3)
C6—C7—C8—C9	2.2 (5)	C8—C9—C18—O1	118.2 (3)
O2—C8—C9—C10	176.5 (2)	C10—C9—C18—O1	−47.3 (3)
C7—C8—C9—C10	0.9 (4)	C8—C9—C18—C19	−61.8 (3)
O2—C8—C9—C18	10.9 (4)	C10—C9—C18—C19	132.6 (3)
C7—C8—C9—C18	−164.7 (3)	O1—C18—C19—C20	−20.0 (4)
C4—C5—C10—C9	−174.9 (3)	C9—C18—C19—C20	160.0 (3)
C6—C5—C10—C9	4.0 (4)	O1—C18—C19—C24	157.6 (3)
C4—C5—C10—C1	4.8 (4)	C9—C18—C19—C24	−22.4 (4)
C6—C5—C10—C1	−176.3 (3)	C24—C19—C20—C21	−0.8 (5)
C8—C9—C10—C5	−4.0 (4)	C18—C19—C20—C21	176.8 (3)
C18—C9—C10—C5	161.3 (2)	C19—C20—C21—C22	−0.7 (5)
C8—C9—C10—C1	176.4 (2)	C20—C21—C22—C23	1.9 (5)
C18—C9—C10—C1	−18.3 (4)	C20—C21—C22—Br2	−177.1 (3)
C2—C1—C10—C5	−3.4 (4)	C21—C22—C23—C24	−1.5 (5)
C11—C1—C10—C5	166.7 (2)	Br2—C22—C23—C24	177.5 (2)
C2—C1—C10—C9	176.2 (2)	C20—C19—C24—C23	1.3 (4)
C11—C1—C10—C9	−13.7 (4)	C18—C19—C24—C23	−176.3 (3)
C2—C1—C11—O3	117.2 (3)	C22—C23—C24—C19	−0.2 (4)

### Hydrogen-bond geometry (Å, °)

**Table:**

*D*—H···*A*	*D*—H	H···*A*	*D*···*A*	*D*—H···*A*
C25—H25B···O1^i^	0.96	2.42	3.313 (4)	155

Symmetry codes: (i) *x*−1, *y*, *z*.
